# The relationship between the psychological resilience and post-traumatic growth of college students during the COVID-19 pandemic: a model of conditioned processes mediated by negative emotions and moderated by deliberate rumination

**DOI:** 10.1186/s40359-024-01853-z

**Published:** 2024-06-19

**Authors:** Yanhua Xu, Yonghui Ni, Jiayan Yang, Jiamin Wu, Yating Lin, Jialu Li, Wei Zeng, Yuqing Zeng, Dongtao Huang, Xingrou Wu, Jinlian Shao, Qian Li, Ziqi Zhu

**Affiliations:** 1 School of Geography and Environment, Jiangxi Normal University, Nanchang, China; 2 School of Geography, South China Normal University, Tianhe District, Guangzhou, 510631 China; 3 Office of International Cooperation and Exchange, Dongguan University of Technology, Dongguan, China

**Keywords:** Psychological resilience, Post-traumatic growth, Negative emotions, Deliberate rumination, Moderated mediation model, COVID-19

## Abstract

**Background:**

The mental health of university students during the COVID-19 pandemic has attracted the attention of researchers. For the present study researchers constructed a mediation model to explore the relationship between psychological resilience and post-traumatic growth, the mediating role of negative emotions and the moderating role of deliberate rumination in students.

**Methods:**

The Psychological Resilience Scale, Posttraumatic Growth Inventory, Depression-Anxiety-Stress Scale (DASS-21) and Event Related Rumination Inventory were used in a survey of 881 college students. The data were analyzed using SPSS 26.0 and the PROCESS plugin (version 3.3).

**Results:**

(1) Psychological resilience is positively related with post-traumatic growth. Deliberate rumination is positively related to psychological resilience, posttraumatic growth, and negative emotions. Psychological resilience, post-traumatic growth and negative emotions are negatively related. (2) Negative emotions mediated the relationship between psychological resilience and post-traumatic growth. (3) Deliberate rumination plays a moderating role in psychological resilience affecting negative emotions. Deliberate rumination plays a moderating role in the extent to which psychological resilience influences PTG through negative emotions.

**Conclusions:**

Psychological resilience affects post-traumatic growth directly and also indirectly through negative emotions. With the increase of mental resilience, the level of negative emotion tended to decrease. When individuals are experiencing negative emotions, high levels of active rumination are more likely to promote post-traumatic growth. This study helps to explore the factors affecting the mental health of college students during the epidemic, thus providing guidance for appropriate mental health interventions.

## Background

The novel coronavirus pneumonia (COVID-19) is a global health threat, and its spread has had a huge impact on the mental health of people around the world. Its rapid development, the speed of spread and the number of people infected has so far taken on unprecedented proportions in more than 150 countries [1]. Global public health and social systems are collapsing under the strain of the coronavirus, and governments have taken extreme public health measures [2] that have caused widespread disruption to social structures and economic activity [3]. Restrictive measures such as quarantines can affect mental health and emotional responses to the pandemic itself [4–6].

Recently published studies have shown that psychological problems such as anxiety and depression are common during epidemics. For example, at the beginning of the COVID-19 outbreak in China, more than half of the population rated its psychological impact as moderate to severe, and about a third of participants reported moderate to severe anxiety [7]. Similarly, prevalence rates of depression and anxiety in North America ranged from 44.1% to 47.2% respectively in Canada [8] to 24.4% and 28.2% in the United States [9]. Similar negative effects on mental health were reported in other countries [10–13].

University students are among the groups whose mental health has been most severely affected by the pandemic [14–16]. Relevant studies have found that the overall prevalence of depression among these students was 23.8% [17], and the proportion of anxiety 24.9%, of which 0.9% was considered severe [18]. A reasonable explanation for this state of affairs is that students were under pressure from both the pandemic and their studies, making them susceptible to a mental disease [19–21].

Social media is one of the main channels for receiving up-to-date information about COVID-19 [22], and while students enjoy its convenience, they may also suffer from information overload, which can cause mental health problems [23]. Due to the pandemic, Chinese educational institutions turn to online instruction, and the lack of learning self-discipline at home has a negative impact on academic achievement, which may have increased depression, anxiety and stress [7]. A number of studies have also shown differences between male and female students with regard to resilience, perception, recovery and growth in the face of trauma [16]. For these reasons, during the COVID-19 outbreak it was important to increase awareness of mental health issues among university students and to adopt positive strategies to address these.

Research has shown that psychological resilience can be a strategy for addressing the mental health challenges posed by the COVID-19 pandemic [24]. This personality trait, explained more comprehensively below, can help individuals cope with the negative psychological effects of traumatic events, including the COVID-19 pandemic [25].

In the last 20 years, with the rise and development of positive psychology, researchers have found that, in addition to exhibiting symptoms of stress, individuals experiencing trauma can derive benefit and experience growth [26]. Research results that link psychological resilience and post-traumatic growth (PTG) have been available for some time; however, the relationship between these two phenomena is contested. It has been suggested that individuals with high psychological resilience scores have low levels of PTG [27] because, although they may experience emotional pain and behavioral or health changes, these are temporary in nature. Their high level of resilience makes them less reactive to traumatic events, and the events are not sufficiently shocking to their existing cognitive schemas, so PTG is less likely to occur [28]. However, other research has shown that high levels of psychological resilience can enhance PTG [29].

The existing literature suggests that negative emotions may be an important mediating variable between psychological resilience and PTG. Such emotions have often been found to be associated with both variables [30, 31]; however, few studies have focused on the possible mediating role of negative emotions between psychological resilience and PTG. In addition, the process of deliberate rumination may contribute to the process of psychological recovery when individuals make sense of their emotions and explore the meaning of the causes and consequences of negative events [32]. Therefore, when individuals experience negative emotions after a traumatic event, their level of psychological resilience and the interaction between PTG, ruminative thinking and future mental health merit further study.

The aim of this study was to investigate the relationship between psychological resilience and PTG, and the mediating role of negative emotions between them, with deliberate rumination included as a moderating factor in the model. As well, gender, a factor that may significantly influence psychological resilience and negative emotions, was added as a covariate and was controlled for in the process.

## Theories and hypotheses

### Psychological resilience

Individual responses to stress and trauma vary greatly, and so people may react differently to the emotional distress caused by a traumatic event such as the COVID-19 pandemic [33]. Psychological resilience, as defined by the American Psychological Association in 2016, is “the process of adapting well in the face of adversity, trauma, tragedy, threats, and even major stressors” [34] (p. 2). This personality trait enables individuals to cope better with stressful or traumatic events and plays an important role in overcoming the adverse effects of stressful environments [35]. In sport, for example, where athletes must overcome difficult challenges in order to succeed, psychological resilience enables individuals to adapt to unfavorable conditions [36]. Although psychological resilience is difficult to measure, previous research has shown that people with higher levels of psychological resilience tend to achieve better mental-health outcomes (e.g., with depression, anxiety and post-traumatic stress disorder) following natural and human-made disasters [37]. Preliminary evidence of the general psychological resilience of adults can be found in studies such as those on people’s bereavement [38, 39] and the terrorist attacks on the World Trade Center in New York City, USA, in 2001 [40]. Therefore, during the novel coronavirus pneumonia, increasing psychological resilience should be a primary public-health priority.

### Post-traumatic growth

A growing number of studies have shown that PTG may be a positive outcome following traumatic events. The definition of trauma in the Diagnostic and Statistical Manual of Mental Disorders considers that “the person has been exposed to a traumatic event in which both of the following were present: (1) the person experienced, witnessed, or was confronted with an event or events that involved actual or threatened death or serious injury, or a threat to the physical integrity of self or others and (2) the person’s response involved intense fear, helplessness, or horror.” [41] People facing a major life crisis (either direct or indirect) often experience distressing emotions, and post-traumatic growth is accompanied by attempts to adapt to highly negative circumstances [42]. Tedeschi and Calhoun [26] suggested that PTG has the following four characteristics: (a) the traumatic event is shocking; (b) the psychological aspect of the struggle with the traumatic event is experienced as positive; (c) the individual’s level of functioning in at least one area is higher than it was before the traumatic event; and (d) growth and psychological distress co-exist [27]. The introduction of the concept of PTG has gradually led to a new perspective on stress and trauma research—a positive growth orientation with a focus on PTG [43]. The COVID-19 pandemic has created an environment in which survival can be in doubt. A more vulnerable environment leads to fear or increased fear of non-survival [44]. Based on the definition of trauma, the COVID-19 pandemic is similar to a profound existential crisis or traumatic experience. How university students coped with and adjusted to this experience, and whether they were able to carry on and grow as a result, is a key research question. Studies have pointed out that there are significant gender differences in the prevalence of post-traumatic stress symptoms (PTSS) [45].

Other existing research shows that psychological resilience is positively correlated with PTG. High levels of psychological resilience can contribute to PTG [29]. Optimism, one dimension of psychological resilience, has been shown to be moderately correlated with PTG [26]. Most people are likely to be exposed to traumatic experiences such as interpersonal violence, death of a loved one, natural disasters, serious industrial or other accidents, war and terrorism to some extent [46–50]. Although these experiences may affect mental health and lead to conditions such as post-traumatic stress disorder [51], the vast majority of individuals exposed to violent or life-threatening events do not develop this disorder. An individual’s level of psychological resilience is a major reason for this outcome. The relationship between psychological resilience and PTG among college students during the COVID-19 outbreak merits attention. Therefore, we propose the following hypothesis:

H1: The psychological resilience of college students has a positive correlation with PTG.

### Negative emotions

Depression, anxiety and stress are risk factors that affect mental health. Previous studies have also found that 28.6% of Chinese students have symptoms of depression [52], and 59.2% have academic stress [53]. In addition, 7.6% of Chinese people suffer from life-long anxiety disorders [54]. There is a close relationship between depression, anxiety and stress [55], and Henry & Crawford believe that the three phenomena can be combined into a higher-order psychological variable called negative emotions [56]. These negative emotions are strongly associated with negative outcomes such as suicidal behavior [57, 58]. and they are negatively correlated with quality of life [59, 60].

Psychological resilience is strongly associated with negative emotions. Research has shown that negative emotions such as anxiety and depression have a negative impact on the development of psychological resilience [61, 62], but some researchers have linked psychological resilience to better mental health outcomes, suggesting that higher psychological resilience predicts lower levels of depression, anxiety and stress [63, 64] and that psychological resilience reduces the likelihood of developing depression [31]. Other researchers consider resilience to be a facilitator of well-being [65] and an indicator of adolescent development [66]. College students are in a period of rapid physical and psychological development, and only a resilient psyche can cope with the negative emotions of depression, anxiety and stress in everyday life. The role of psychological resilience in regulating and associating negative emotions among college students is particularly important in situations where COVID-19 has triggered a variety of lifestyle changes that also lead to a high incidence of negative emotions. Therefore, we propose the following hypothesis:

H2a: Psychological resilience among college students is negatively associated with negative emotions.

A moderately positive relationship between negative emotions and PTG has been reported [67]. The model of PTG proposed by Tedeschi and Calhoun [42] suggests that events leading to psychological stress stimulate cognitive processing, and that when such thinking is constructive individuals think positively about their post-traumatic self, others and the world, contributing to PTG. This means that the individual experiencing negative emotions may also experience PTG. Negative life events such as the COVID-19 outbreak, inevitably lead to negative emotions among college students, but whether this results in PTG has been less studied and reported on. Therefore, the following hypotheses is proposed:

H2b: Negative emotions of college students have a positive correlation with PTG.

On the basis of the literature and the three hypotheses mentioned above, we further propose the following hypothesis:

H2c: Negative emotions of university students act as a mediator between psychological resilience and PTG.

### Deliberate rumination

After a traumatic event, and especially after a global traumatic event like COVID-19, individuals may revisit or reconsider their beliefs. This cognitive process is referred to as rumination [68]. There are many different classifications of ruminant, and the classification method of Tedeschi et al. [42] will be adopted in this paper. He categorized ruminant into intrusive rumination and deliberate rumination. Intrusive rumination is a non-constructive cognition process whereby a traumatic event passively intrudes into an individual’s cognition, and they ponder negative states and emotions in an evaluative manner. Conversely, deliberate rumination is a constructive cognition process whereby the individual consciously thinks positively about the cues related to the traumatic event, tending to face the dilemma openly and solve the problem. The following is a list of some of the most important features.

Researchers have also begun to explore the relationship between rumination and psychological resilience, suggesting that deliberate rumination, which involves making sense of one’s emotions and exploring the causes and consequences of negative events, may contribute to the process of psychological recovery [69]. Those with high levels of psychological resilience are more likely to focus on and process positive information and the positive aspects of their personal experiences [70] and therefore have higher levels of deliberate rumination. As well, psychological resilience and rumination were included when factors that influence recovery from post-traumatic stress were explored [71], with psychological resilience considered as facilitating recovery and rumination as hindering it. However, the relationship between these two variables has been the subject of little discussion. Therefore, we propose the following hypothesis:

H3a: The psychological resilience of college students has a positive correlation with deliberate rumination.

Research has consistently found that stress, anxiety, depressive symptoms and other negative emotions are associated with rumination. For example, cross-sectional studies have shown that people who are more prone to negative emotions have stronger negative rumination and weaker positive rumination than normal people [72]. More important, longitudinal studies have found that negative emotions are associated with increased rumination [73, 74]. In one study, the relationship between rumination and anxiety was significant, with a correlation coefficient of 0.32 [75]. However, deliberate rumination, which represents a constructive form of deep thinking, may relate differently with negative emotions than in previous studies. Therefore, the following hypothesis is proposed for this study.

H3b: Negative emotions in college students has a negative correlation with deliberate rumination.

On the basis of the literature and the above two hypotheses, we further propose the following hypothesis:

H3c: The interaction of psychological resilience and deliberate rumination has a negative correlation with negative emotions.

Researchers have argued in the literature that deliberate rumination can promote PTG [68, 76, 77]. PTG is the positive result of struggling with and coping with a traumatic event, and cognitive processing plays a key role in its development [42]. Deliberate rumination enables a person to actively reflect on and reevaluate their cognitive processes and ways of thinking after trauma and to shift their personal attention to its positive aspects [78]. Therefore, we propose the following hypothesis:

H4a: Deliberate rumination by college students has a correlation with PTG.

On the basis of the literature and the two hypotheses presented above, we further propose the following hypothesis:

H4b: The interaction of negative emotions and deliberate rumination has a positive correlation with PTG.

**Fig. 1:**
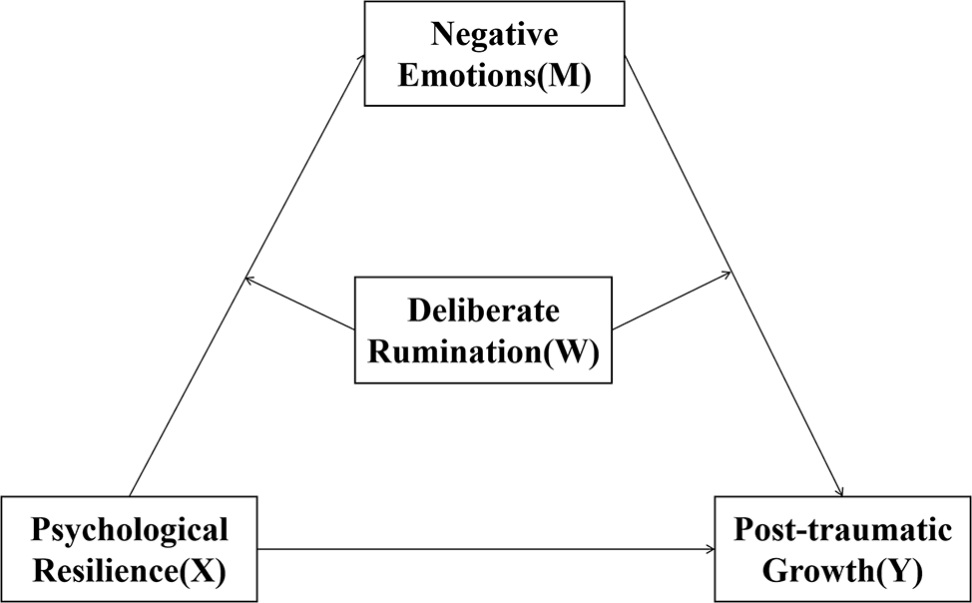
Moderated mediation model of the present research

In summary, based on the relationship between psychological resilience and PTG, we propose a moderator-mediator model (see Fig. 1). Our aim is to provide insight into how (in the mediating role of negative emotions) and under what circumstances (the mediating role of deliberate rumination) psychological resilience leads to higher levels of PTG.

## Materials and methods

### Participants and procedure

The study was carried out at an independent college in Guangdong Province, China. The college has more than 20,000 undergraduate and specialist students in 44 disciplines. Here is the formula to determine the sample size:

$$n=\frac{{Z}^{2}p(1-p)}{{e}^{2}} \left(1\right)$$

n is the sample size, Z is the standard error, p is the variance of the population estimate, and e is the permissible error.

When the 95% confidence interval, Z is 1.96. p is taken to be 0.5 for prudent estimation. The formula calculation shows that at least 385 participants are needed for this study. A total of 918 students completed a questionnaire. Questionnaires for non-Guangdong students were removed from the data, reducing the sample size to 881. Sample size is higher than 385.This method of data selection is to better understand the relevant situation of students in epidemic areas and make this study more targeted. The respondents comprised 317males (35.982%) and 564 females (64.018%), all from a single year. To generate research ideas and hypotheses, exploratory focus-group interviews were conducted at the college before the research design was finalized. Interviews were convened by the course teacher and conducted online. The majority of the interviewees indicated that they had experienced negative emotions for a variety of reasons during COVID-19. Some students also said that they could better regulate their emotions when they had negative emotions because of their high ability to resist pressure. Other students said that thinking about positive things can help them recover and even gain something.

Data were collected for this study between April 10 and June 15, 2020 using an online questionnaire survey. The QR code for the questionnaire was presented to students enrolled in same-level English classes during a break. (In China, QR codes are widely used for such tasks as information retrieval, mobile payments and internet links, and they are informative, easy to identify and cost-effective.) The study received an ethical review from the School of Geographic Sciences, South China Normal University. Before students filled out the questionnaire, we asked “Do you agree to participate in this survey?” online to investigate the willingness of the students to participate in the survey, with students ticking “1” if they agreed and “2” otherwise. The study was explained to them after obtaining their consent to participate in the survey and the students completed the questionnaire on a voluntary basis.

### Measures

The questionnaire used in this study consisted of five sections: (a) demographic data, (b) Psychological Resilience Scale, (c) Post-Traumatic Growth Inventory, (d) Depression, Anxiety and Stress Scale –DASS-21, and (e) Event Related Rumination Inventory. The demographic information section included gender and current place of residence. To check the accuracy of the existing Chinese version, the English versions of the scales were translated into Chinese using a back-translation method [79]. One researcher translated the English versions into Chinese, then another researcher translated the Chinese versions back into English, and finally a third researcher compared the original, translated and back-translated versions to check for equivalence between the original English versions and the translated Chinese versions. Any non-equivalence was resolved prior to data collection. All scales selected for this study had been used in other studies in the Chinese context and had good reliability and validity [80–83].

### Psychological resilience scale

The Psychological Resilience Scale used in this study was proposed by Hu and Gan [80] after reviewing conceptualizations of psychological resilience proposed by Chinese and foreign scholars [84]. The scale’s 27 items are divided into five dimensions: (a) goal focus (5 items), (b) emotional control (6 items), (c) positive perception (4 items), (d) family support (6 items) and (e) interpersonal assistance (6 items). A 5-point Likert rating is used:0.1 = completely disagree, 2 = somewhat disagree, 3 = unsure, 4 = somewhat agree, and 5 = completely agree. The theoretical score ranges from 27 to 135. The higher the score, the greater the psychological resilience of the participant. The Cronbach α coefficient for this scale was 0.860 and its 95% confidence intervals were [0.836,0.879].

### Posttraumatic growth inventory

This study used the Post-Traumatic Growth Inventory, originally proposed by Tedeschi and Calhoun [26, 30] and translated into Chinese by Geng et al. [81] to suit the Chinese context. The 21-item scale is divided into five dimensions: (a) interpersonal relationships (7 items), (b) new possibilities (5 items), (c) personal strengths (4 items), (d) spiritual change (2 items) and (e) appreciation of life (3 items). A 6-point Likert scale is used: 1 = never; 2 = few; 3 = a few; 4 = medium; 5 = more; 6 = most. The theoretical score ranges from 21 to 126. The higher the score, the higher the degree of post-traumatic growth. The Cronbach α coefficient for this scale was 0.958 and its 95% confidence intervals were [0. 955,0.961].

#### Depression-anxiety-stress scale 21 – DASS-21

The Depression-Anxiety-Stress Scale 21 – DASS-21 used in this study was revised by Lovibond, which has 21 items divided into three dimensions: depression, anxiety and stress [85]. Each dimension has 7 items. The Likert scale used has 4 points: 1 = not conforming, 2 = somewhat conforming, 3 = often conforming, 4 = always conforming. The theoretical score ranges from 21 to 84. The higher the score, the higher the frequency of the depression, anxiety and stress conditions. The Cronbach α coefficient for this scale was 0.960 and its 95% confidence intervals were [0.956,0.963].

### Event related rumination inventory

The study used the “Deliberate Rumination” section of the Event-Related Rumination Scale proposed by Cann et al. in 2011 [68], which consists of 10 items. A 4-point Likert rating is used: 1 = not at all, 2 = sometimes, 3 = often, and 4 = always. The theoretical score ranges from 10 to 40. The higher the score, the higher the frequency of deliberative rumination. The Cronbach α coefficient of this scale was 0.913 and its 95% confidence intervals were [0.903,0.920].

### Data analysis

In this study, statistical analysis of the data was mainly carried out using SPSS 26.0 statistical software. Firstly, Harman’s single-factor test was used to test for common method bias [86]. Secondly, a series of descriptive analyses were carried out to test for trends in concentration and dispersion of the data. Pearson product moment correlation coefficients were then calculated to test the relationships between the independent, mediator, moderator and dependent variables. Finally, moderator-mediator models of psychological resilience and PTG were tested using the PROCESS plugin (version 3.3) in SPSS, developed by Hayes specifically for path analysis-based moderator and mediator analyses and their combinations [87]. Bias-corrected 95% confidence intervals for conditional direct and indirect effects were estimated from 5,000 data resamples, and the effects were significant when the confidence intervals did not include zero [75, 88]. In addition, gender was used as a control variable in this study.

## Results

### Testing for common method bias

In order to ensure the validity of the data analysis, the common method bias (CMB) was first investigated by the Harman single factor test. CMB refers to the fact that the use of the same type of data source, the same measurements or the same data collection environment may result in artificial covariances between association and criterion variables and impose an unrealistic relationship between them [86, 89]. As all data in this study were collected using self-report questionnaires, in the same environment and over the same time period, this source of bias needed to be controlled and tested.

**Fig. 2:**
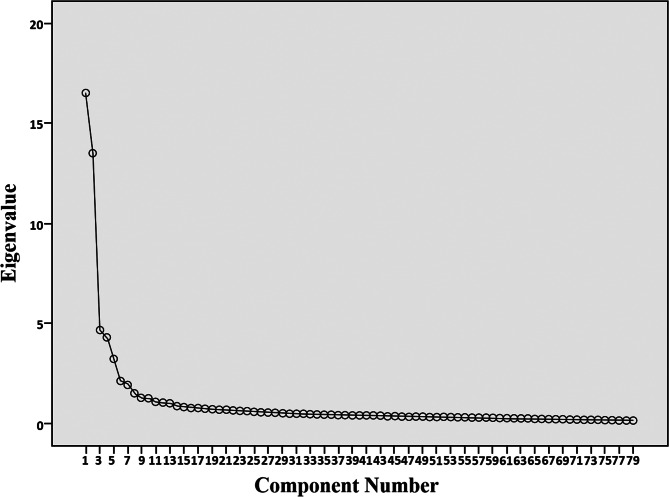
Scree plot

The Harman single-factor test was used to carry out principal component analysis of all 79 items in the questionnaire (except demographic variables). The results showed that 15 components had eigenvalues greater than 1. The contribution of the 15 components to the total variance was 64.735%. As can be seen from the Scree plot (Fig. 2), the curve tends to be stable after the eighth component, and the contribution rate of the eight components to the total variance is 60.252%. The first component accounting for only 20.868%, well below 40%, which is the cut-off value for classifying the magnitude of common method deviations [90]. Therefore, it can be stated that there was no significant common method bias in this study. In other words, the covariance between the dependent and independent variables in this study is largely attributable to their nature rather than to the measurement methods used in the data-collection process.

### Descriptive and correlation analysis

Table 1 summarizes the results of the descriptive and correlational analyses of the four variables in this study. The study showed that the psychological resilience of college students was significantly and positively correlated with PTG, deliberate rumination (*r* = 0.414, *p* < 0.01; *r* = 0.077, *p* < 0.05) and negatively correlated with negative emotions (*r*=-0.469, *p* < 0.01). PTG was significantly positively correlated with deliberate rumination (*r* = 0.353, *p* < 0.01) and negatively correlated with negative emotions (*r* = -0.081, *p* < 0.05). Deliberate rumination was also significantly and positively correlated with negative emotions (*r* = 0.158, *p* < 0.01).

**Table 1:** Mean, standard deviation and correlations between variables

Variables	M	SD	1	2	3	4
1. Psychological Resilience	3.417	0.443	1			
2. Post-Traumatic Growth	3.302	1.002	0.414**	1		
3. Negative Emotions	1.626	0.562	-0.496**	-0.081*	1	
4. Deliberate Rumination	2.012	0.520	0.077*	0.353**	0.158**	1

Note. *N* = 881

*p<0.05, **p<0.01 (2-tailed)

### Testing the mediation model

The mediation analysis was conducted using psychological resilience as the independent variable, PTG as the dependent variable and negative emotions as the mediating variable (model 4) in PROCESS (version 3.3). The results, summarized in Table 2, showed that gender had a certain impact on students’ psychological resilience and negative emotions(β=-0.073, *p* < 0.05). Psychological resilience positively associated PTG (β = 1.127, *p* < 0.001), after controlling for gender. Psychological resilience negatively associated negative emotions (β=-0.623, *p* < 0.001), whereas negative emotions positively associated PTG (β = 0.289, *p* < 0.001). The total effect of psychological resilience on PTG was 0.946 (95% CI = [0.810, 1.083]) as tested by the bias-corrected percentile bootstrap method, with direct (95% CI = [0.972, 1.282]) and indirect (95% CI = [-0.270, -0.093]) effects. These results mean that psychological resilience can have a direct impact on PTG or an indirect impact on PTG through negative emotions.

**Table 2:** Analysis of the mediation effect

Independent Variable	STEP1			STEP2			STEP3		
	Post-Traumatic Growth			Negative Emotions			Post-Traumatic Growth		
	β	SE	t	β	SE	t	β	SE	t
Gender	-0.110	0.064	-1.715	-0.073	0.034	-2.126*	-0.089	0.064	-1.399
Psychological Resilience	0.946	0.070	13.607***	-0.623	0.037	-16.763***	1.127	0.079	14.262***
Negative emotions							0.289	0.062	4.632***
R^2^	0.175			0.250			0.194		
F	92.807***			146.320***			70.466***		

Note. *N* = 881

*p<0.05, **p<0.01, ***p<0.001 (2-tailed)

### Testing the moderated mediation model

After adding moderator (deliberate rumination) to the first and second halves of the mediation model, Model 58 was used to analyze the moderated mediation model. The results showed (see Table 3) that gender differences exist between the variables in this model (β=-0.091, *p* < 0.05; β=-0.153, *p* < 0.05). The correlation of psychological resilience and deliberate rumination was statistically significant in affecting negative emotions (β = -0.155, t = -2.546, *p* < 0.001). This suggests that deliberate rumination can play a moderating role in psychological resilience affecting negative emotions. Similarly, the correlation of negative emotions and deliberate rumination was also statistically significant in affecting PTG (β = -0.297, t = -3.822, *p* < 0.001), suggesting that deliberate rumination also plays a moderating role in the extent to which psychological resilience influences PTG through negative emotions.

**Table 3:** Moderated mediation test

Independent Variable	STEP1			STEP2		
	Negative Emotions			Post-Traumatic Growth		
	β	SE	t	β	SE	t
Gender	-0.091	0.033	-2.734*	-0.153	0.060	-2.564*
Psychological Resilience	-0.637	0.036	-17.594***	0.961	0.075	12.756***
Deliberate Rumination	0.217	0.031	7.037***	0.639	0.057	11.131***
Psychological Resilience* Deliberate Rumination	-0.155	0.061	-2.546*			
Negative Emotions				0.145	0.060	2.411*
Negative Emotions* Deliberate Rumination				-0.297	0.078	-3.822***
R2	0.296			0.297		
F	92.080***			73.840***		

Note. Analyses conducted using PROCESS model 14. *N* = 881

*p<0.05, **p<0.01, ***p<0.001

We further analyzed the regulatory role of deliberate rumination using a simple slope test. The adjustment variables were grouped according to the mean score of deliberate rumination plus or minus one standard deviation. The mean plus one standard deviation was designated as the high deliberate rumination group, while the mean minus one standard deviation was designated as the low deliberate rumination group. The analysis leads to the following conclusions:

- When the level of deliberate rumination was low, as the level of psychological resilience increased, the level of negative emotion showed a tendency to decrease **(Effect=-0.555, t=-11.238, ***p*** < 0.001).**
- When the level of deliberate rumination was high, as the level of psychological resilience increased, the level of negative emotion showed a more significant tendency to decrease than the low level. **(Effect=-0.717, t=-15.300, ***p*** < 0.001).**
- When the level of deliberate rumination was low, as the level of negative emotions increased, the level of post-traumatic growth significantly increased **(Effect = 0.300, t = 4.099, ***p*** < 0.001).**
- When the level of deliberate rumination was high, as the level of negative emotions increased, there was no significant change in the level of post-traumatic growth **(Effect=-0.009, t=-0.129, ***p***>0.5).**

The above results suggest that after controlling for gender variables, deliberate rumination played a significant moderating role in the relationship between psychological resilience and negative emotion, and negative emotion and post-traumatic growth.

**Table 4:** Conditional indirect effect at specific levels of deliberate rumination when mediated by psychological resilience

Moderator Variable	Effect	se	t	95%CI	
				LLCI	ULCI
M-1SD	-0.555	0.049	-11.238	-0.653	-0.459
M	-0.634	0.036	-17.594	-0. 708	-0.566
M + 1SD	-0.717	0.047	-15.300	-0.810	-0.626

**Table 5:** Conditional indirect effect at specific levels of deliberate rumination when mediated by negative emotion

Moderator Variable	Effect	se	t	95%CI	
				LLCI	ULCI
M-1SD	0.299	0.073	4.094	0.156	0.443
M	0.145	0.06	2.411	0.027	0.263
M + 1SD	-0.01	0.072	-0.134	-0.150	0.131

**Fig. 3:**
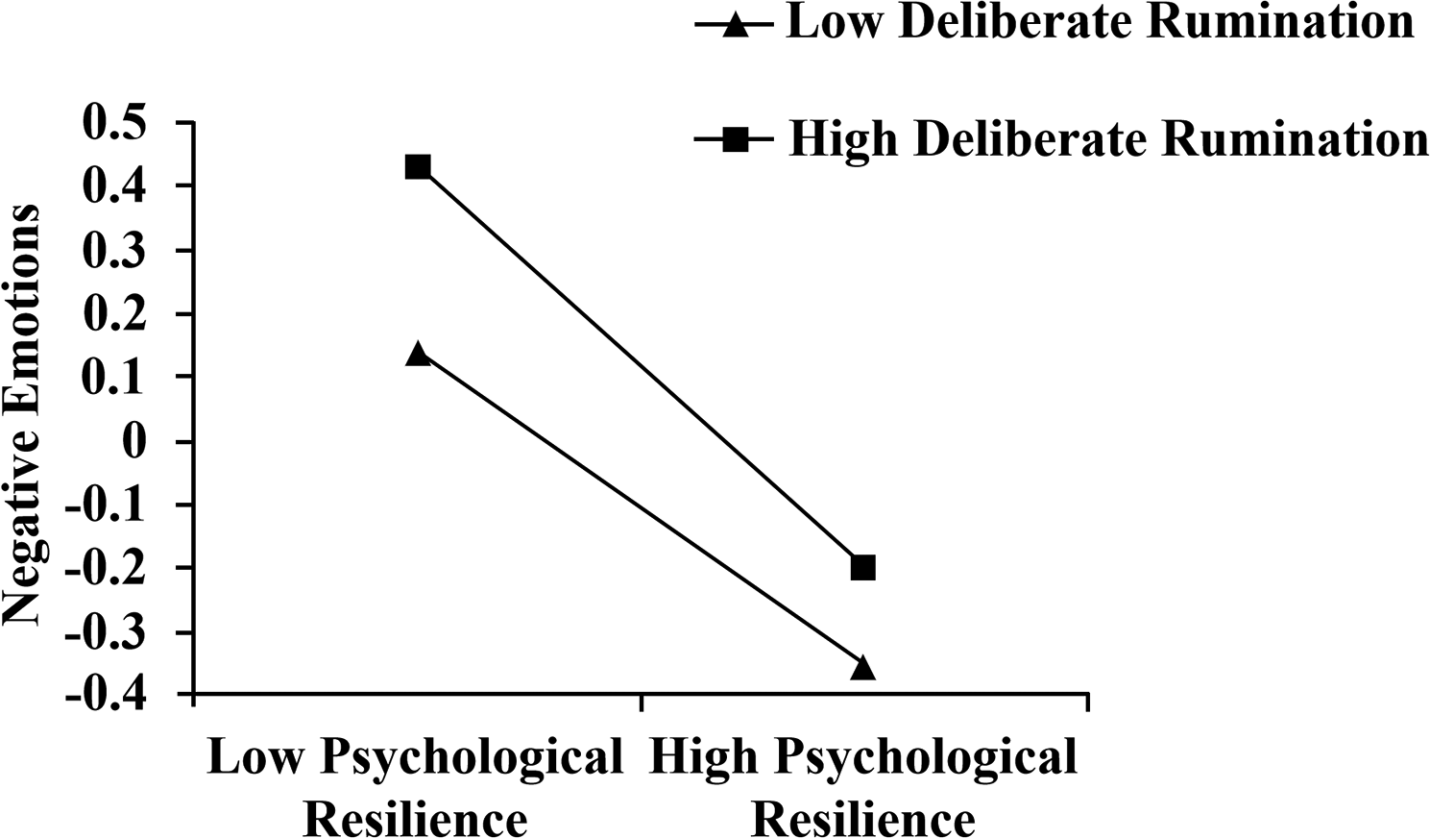
The relationship between psychological resilience and negative emotion for high and low levels of deliberate rumination

**Fig. 4:**
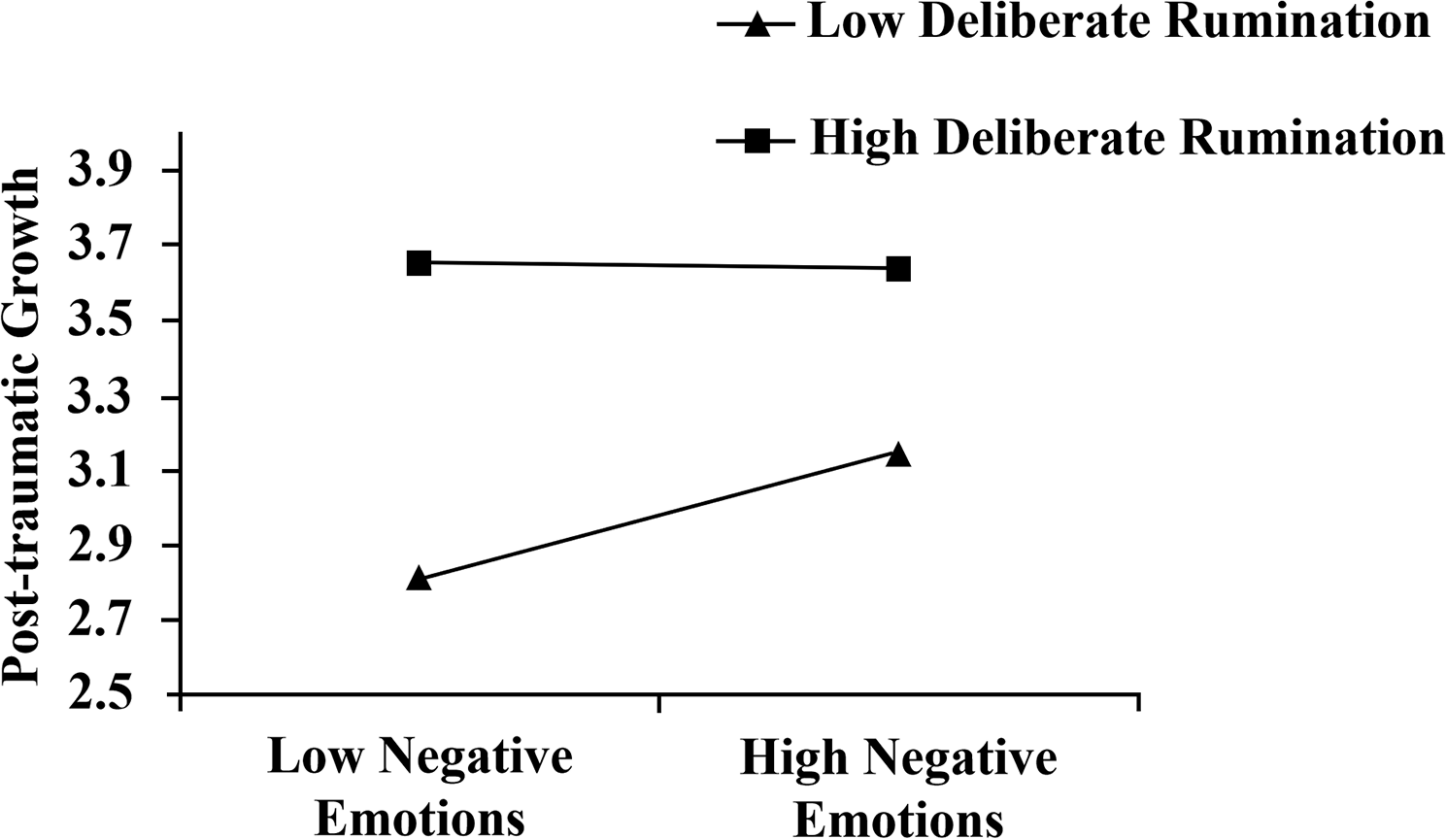
The relationship between negative emotion and post-traumatic growth for high and low levels of deliberate rumination

**Fig. 5:**
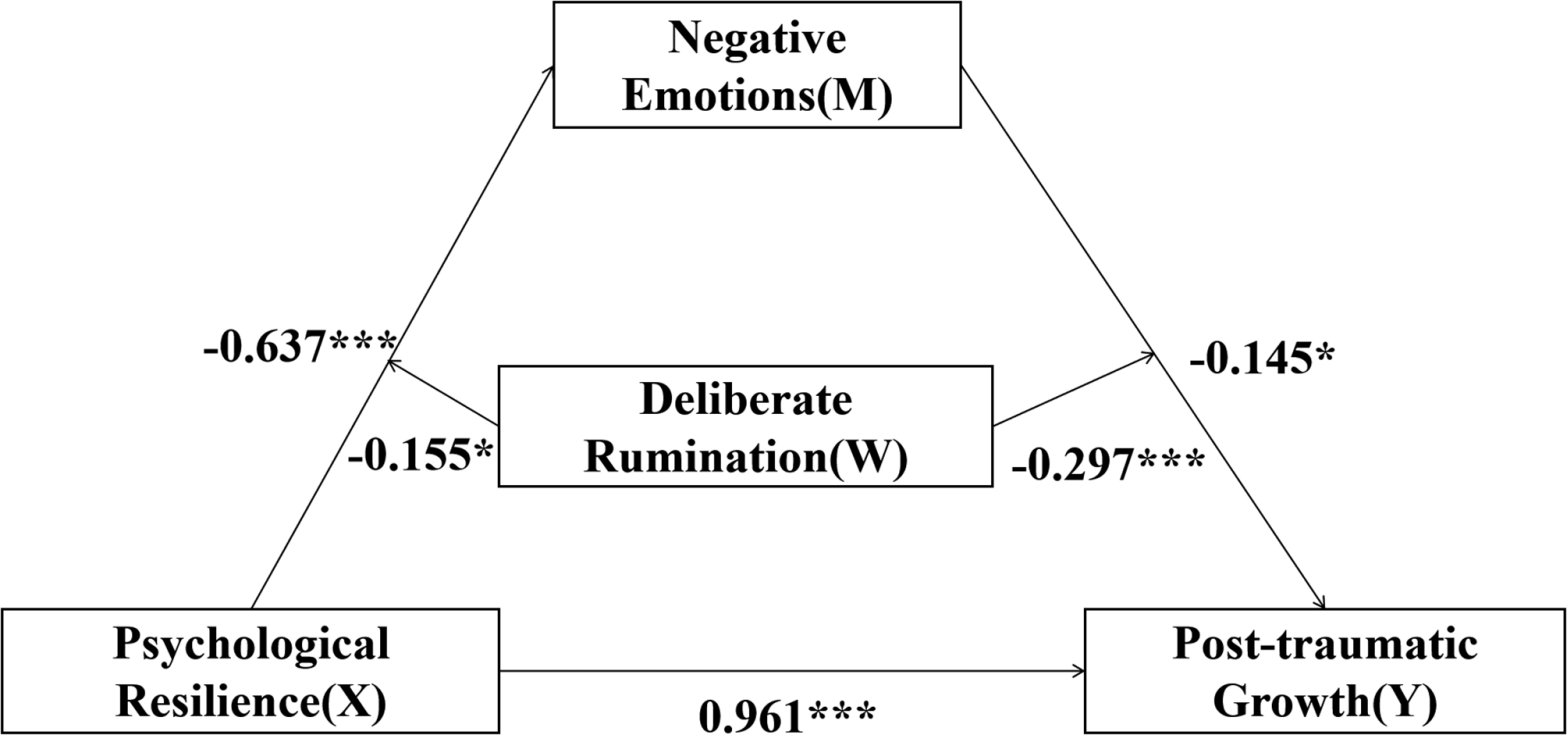
**T**he standardized estimate values of the final model

## Discussion

### Discussion of the results

The results are generally consistent with the hypotheses offered in the present study and the conclusions of previous studies, although the hypotheses related to deliberate rumination were not confirmed. According to the literature, exposure to public-health emergencies (such as the Ebola epidemic [91], earthquakes [92] and SARS [93]) has caused mental-health problems. The COVID-19 pandemic and its preventive measures have also greatly affected mental health. Early studies of the COVID-19 outbreak reported significant psychological effects from both the outbreak and the response, suggesting that study participants exhibited high levels of psychopathological symptoms [7]. Furthermore, gender is a consistent factor affecting psychological outcomes: women are relatively more vulnerable to psychological distress than men, exhibiting moderate levels of anxiety [7, 14]. It has also been noted that there are significant gender differences in the prevalence of post-traumatic stress symptoms (PTSS) [45]. The results of the present study suggest that females are associated with increased anxiety, depression and stress. This finding is consistent with previous research findings [94].

Another possible explanation for poorer mental health during the COVID-19 outbreak is related to information overload [95]. A recent study in mainland China found that the higher the frequency of social media exposure, the higher the likelihood of anxiety [23].

First, these results are consistent with H1 and also with the results of other studies that confirmed the existence of a relationship between psychological resilience and PTG [96, 97]. College with greater psychological resilience reported higher levels of PTG [29, 30]. According to relevant studies, resilience has been widely studied in other populations including patients, political refugees and survivors of natural disasters, which previously highlighted strong psychological resilience can help person better endure trauma during the event, recover and maintain more positive mental health state after the event [98–100]. Psychological resilience is associated with numerous desired health outcomes [101, 102]. Colleges with higher psychological resilience have a greater sense of self-control, are more optimistic in their outlook, are stronger and more optimistic in the face of trauma, are more positive about their traumatic experiences, and mobilize their own personal and environmental resources to help them move beyond the trauma. It also depends on individual qualities or skill in coping with adversity navigating and negotiating to resources [103]. In addition, individuals who demonstrate psychological resilience or PTG are significantly more resistant to possible future traumatic events [104].

Second, the findings are consistent with H2a, H2b and those of other studies. In this study, psychological resilience negatively affected negative emotions, similarly to conclusions of Ren and Cai [105]. In our undergraduate participants, we observed that the psychological resilience of college students was a negative association of negative emotions. This is consistent with the findings of Way K. W. Lua, who used cross hysteresis to study the relationship between mental resilience of college students and symptoms of depression and anxiety, and this research showed that psychological resilience of college students negatively affected depressive symptoms in a certain period of time [106]. Psychological resilience has been extensively studied in diverse groups such as adult refugees, patients with depression, and college students without mental illness, but few studies have focused on the mediating effect of negative emotions on mental resilience. However, our findings are consistent with previous emphasis on the potential protective effects of mental resilience on mental health in different populations. Poudel Tandukar K et al. showed in their study that adult refugees with the highest mental resilience had the lowest risk of above-threshold anxiety and depression [99]. Chen, N. et al. believe that anxiety and depression can be alleviated by improving the psychological resilience of patients with depression [107]. These findings suggest that the negative association of mental resilience and negative emotions apply to different groups. Furthermore, negative emotions negatively affect PTG. This is consistent with the findings of narrative analysis by Zieba et al., students in the high PTG group mentioned more positive emotions than negative emotions in the narrative [108]. The degree of negative emotions caused by various traumatic events was relatively high in college students, but negative emotions did not significantly affect PTG [109, 110]. Negative emotions can give individuals negative psychological feelings and hinder their recovery from traumatic experiences, thus affecting the formation of PTG [111]. The findings of other studies have demonstrated that the expression of negative emotions through venting can facilitate the development of PTG at higher levels [112]. Emotional expression and emotional processing can affect PTG in traumatized individuals [113, 114].

Third, the results of this study are consistent with H2c and the results of other studies. These studies suggest that negative emotions play a mediating and buffering role in psychological resilience and PTG. Psychological resilience is positively correlated with mental health [115]. When faced with stressful or adverse situations, those with higher levels of psychological resilience tend to experience lower levels of depression or anxiety and have the ability to recover more quickly to the pre-crisis stage and reach a pre-stress baseline more quickly [116]. However, those with lower levels of psychological resilience have more difficulty coping with the emotional challenges of a pandemic crisis [34]. A study of U.S. adults found that psychological resilience was negatively correlated with the level of concern about COVID-19: individuals with higher levels of psychological resilience were less affected by future anxiety and the perceived threat of COVID-19, and thus experienced less impact on their subjective well-being, than those with lower levels of psychological resilience [117]. Such negative emotional reactions during a disaster can predict the occurrence of mental health problems after the disaster [118]. Therefore, people with higher levels of psychological resilience are able to grow more in response to COVID-19 induced emotional distress [116].

Fourth, the findings contradict hypotheses H3a, H3b, H3c, H4a and H4b. Previous research has shown that for many people coping with a variety of negative emotions in their lives, deliberate rumination on an event involves a process of examination of the event and its meaning [119]. Therefore, students with higher psychological resilience are better able to reduce their negative emotions through deliberate rumination. This is due to the fact that deliberate rumination is a variety of recurrent, event-related thinking that includes understanding the meaning of the event, problem solving, recollection of the event, and anticipation of the future [120]. At the same time, when trauma occurs, individuals with higher levels of deliberate rumination are able to evaluate and interpret the traumatic event rationally, try to discover the meaning embedded in the traumatic event, reconstruct their perceptions of the traumatic event and the world, i.e. they engage in deliberate rumination about the traumatic event, and thus gain post-traumatic growth [121]. The students in this study were still studying at home online during the study period and had been exposed to the traumatic experiences of the epidemic. Therefore, their psychological health problems should be actively intervened to promote the moderating effect of deliberate rumination. And the related mechanisms are yet to be studied in depth in follow-up.

### Implications

The main theoretical implication of this study is the confirmation of the link between psychological resilience and PTG, which deepens the focus on these two phenomena and their interaction in a highly traumatic situation like a pandemic. By analyzing mediating and moderating effects, it was found that higher psychological resilience among college students may lead to a reduction in negative emotions, and then to PTG. Furthermore, deliberate rumination negatively regulates this mediation model, a finding that is inconsistent with previous research findings.

In practical terms, the relationship between the four variables proposed in this study may help to mitigate the occurrence of negative emotions among college students, and subsequently promote their physical and psychological well-being, growth and success during the pandemic.

### Limitations and future directions

There are also some limitations to this study. First, this study used a cross-sectional design. Although cross-sectional studies combined with data analysis can analyze relationships between variables, causality cannot be determined reliably. Future researchers could use a longitudinal survey design to collect data over time. Second, the data were all collected using self-report methods, and although the Harman single-factor test results suggested the absence of common-method bias, future studies could profitably use a combination of parent reports and teacher evaluations to collect data. Moreover, data collection began on April 10, 2020, when PTG was not evident enough. This could be followed up further in future studies. Finally, all participants were from one university, which may undermine the generalizability of the findings. In the future, recruiting participants from different institutions could strengthen generalizability.

## Conclusion

COVID-19 is considered to be among those recent epidemics with the most serious psychosocial impact, and interventions are needed to assist people improve their mental health. This study explored the relationship between psychological resilience and PTG among college students during the COVID-19 pandemic, as well as the mediating role of negative emotions between the two and the moderating role of deliberate rumination in the pre- and post-pathway. The results showed that psychological resilience was positively associated with PTG and that negative emotions partially mediated between psychological resilience and PTG. Furthermore, deliberate rumination affected both the front and back half of PTG by regulating psychological resilience through negative emotions. The mental health and well-being of college students is a major concern for education authorities and universities, and attention to, research on and interventions with the psychological outcomes of university students who have experienced traumatic events are of paramount importance. The results of this study may help us understand the factors that influenced the mental health of college students during the COVID-19 pandemic and thus lead to appropriate mental-health interventions that enable positive change after their traumatic experiences.

## Data Availability

The datasets used and/or analyzed during the current study are not publicly available. Permissions could be obtained/required from the corresponding author on a reasonable request.

## Abbreviations

DASS: 21 Depression-Anxiety-Stress Scale
COVID: 19 Novel coronavirus pneumonia
PTG: Post Traumatic growth
PTSS: Post traumatic stress symptoms
CMB: Common method bias
